# Ascorbic Acid Deficiency Prevalence and Associated Cognitive Impairment in Alcohol Detoxification Inpatients: A Pilot Study

**DOI:** 10.3390/antiox10121892

**Published:** 2021-11-26

**Authors:** Virgile Clergue-Duval, Julien Azuar, Julien Fonsart, Clément Delage, Dorian Rollet, Jihed Amami, Alexia Frapsauce, Marie-Astrid Gautron, Eric Hispard, Frank Bellivier, Vanessa Bloch, Jean-Louis Laplanche, Frank Questel, Florence Vorspan

**Affiliations:** 1 APHP GHU Nord, Site Lariboisière Fernand-Widal, Département de Psychiatrie et de Médecine Addictologique, 75010 Paris, France; julien.azuar@aphp.fr (J.A.); dorian.rollet@aphp.fr (D.R.); jihed.amami@aphp.fr (J.A.); eric.hispard@aphp.fr (E.H.); frank.bellivier@inserm.fr (F.B.); franck.questel@aphp.fr (F.Q.); florence.vorspan@aphp.fr (F.V.); 2 Inserm UMRS-1144 Optimisation Thérapeutique en Neuropsychopharmacologie, Université de Paris, 75006 Paris, France; clement.delage@aphp.fr (C.D.); vanessa.bloch@aphp.fr (V.B.); jean-louis.laplanche@aphp.fr (J.-L.L.); 3 FHU Network of Research in Substance Use Disorders (NOR-SUD), 75006 Paris, France; marieastrid.gautron@aphp.fr; 4 UFR de Médecine, Université de Paris, 75006 Paris, France; 5 Resalcog (Réseau Pour la Prise en Charge des Troubles Cognitifs Liés à L’alcool), 75017 Paris, France; 6 APHP GHU Nord, Site Lariboisière Fernand-Widal, Département de Biochimie et Biologie Moléculaire, 75010 Paris, France; julien.fonsart@aphp.fr; 7 APHP GHU Nord, Site Lariboisière Fernand-Widal, Service de Pharmacie, 75010 Paris, France; alexia.frapsauce@aphp.fr; 8 APHP GHU Nord, Site Beaujon, Unité de Traitement Ambulatoire des Maladies Addictives, 92110 Clichy, France; 9 UFR de Pharmacie, Université de Paris, 75006 Paris, France

**Keywords:** ascorbic acid, cognitive impairment, alcohol use disorder, malnutrition, central nervous system, sedative use disorder, liver cirrhosis, homeless persons, HIV

## Abstract

Malnutrition has been reported in alcohol use disorder patients as having a possible influence on cognitive function. The aim of this study was to analyse the prevalence of ascorbic acid (AA) deficiency in inpatients admitted for alcohol detoxification and the associated factors, including cognitive impairment in the early period of abstinence. A retrospective chart review was conducted. The AA level was categorised into three groups: deficiency (AAD) (<2 mg/L), insufficiency (AAI) (2–5 mg/L) and normal level. The cognitive impairment was screened using the Montreal Cognitive Assessment (MoCA). Ninety-six patients were included (74 men; mean age 49.1 years (±11.5)). Twenty-seven AAD (28.1%) and twenty-two AAI (22.9%) were observed. In multivariate analysis, risk factors for AAD versus normal AA level were men (OR 17.8, 95%CI (1.63–194)), compensated cirrhosis (OR 9.35, 95%CI (1.60–54.6)) and street homelessness (OR 5.76, 95%CI (1.24–26.8) versus personal housing). The MoCA score was available for 53 patients (mean MoCA score: 25.7 (±3.3)). In multivariate analysis, the natural logarithm of AA (β = 1.18, *p* = 0.037) and sedative use disorder (β = −2.77, *p* = 0.046) were associated with the MoCA score. AAD and AAI are frequent in inpatients admitted for alcohol detoxification. A low level of AA was associated with cognitive impairment in the early period of abstinence.

## 1. Introduction

Malnutrition contributes to acute alcohol-related cognitive impairment in patients with alcohol use disorder (AUD) through several mechanisms, not limited to thiamine deficiency or thiamine dysmetabolism [1,2,3]. Those acute cognitive impairments relate to several functions (episodic memory and executive functions) and are observed immediately after alcohol withdrawal, early in abstinence. They are most often transient and usually decrease under appropriate treatment (healthy nutrition, alcohol abstinence and neuropsychological training) [3,4,5] within days or weeks. The relevance of nutritional support in the care of patients with AUD undergoing detoxification is not only intended for the prevention of Wernicke encephalopathy and Korsakoff syndrome. Nutritional support also aims at taking care of the highly prevalent malnutrition observed in this specific population [6] due to altered intakes and variable alterations of nutrient metabolism in AUD patients [7].

Each nutrient deficiency has a different prevalence [1,8]. Regarding ascorbic acid deficiency (AAD), a high prevalence was observed in patients admitted for alcohol detoxification, between 7.5% and 57.4% [1,9,10,11]. Those rates are higher than in the general adult population, where it is estimated at 8.4% [12,13]. However, uncertainties remain about the real prevalence due to heterogeneity in these studies’ design, including small sample size [8,10] or specific settings such as intensive care units [11]. This high prevalence of AA deficiency in patients with AUD may play an important role in the vulnerability of those patients to several pathophysiological processes, as the antioxidant roles of Vitamin C were shown to be protective in cardiovascular diseases, infectious diseases or cancer [14,15]. Additionally, its effect is discussed in cognitive impairment in elderly populations or in patients with neurodegenerative diseases [16,17,18,19]. Because ascorbic acid (AA)’s cognitive protective role could be mediated by its demonstrated effect to attenuate the excitotoxicity of glutamate [19,20,21], which is involved in alcohol withdrawal [3], the impact of AAD could be more pronounced in patients with AUD. In the general population, risk factors for AAD and insufficiency (AAI) were identified. The rate is higher in hospitalised patients [22,23], men [12,13,22,24], current tobacco smokers [12,13,22,24], deprived persons [12,22,24] and the homeless [25]. However, the specific risk factors in AUD patients have never been studied.

This is why we thought that it was of specific interest to search for AAD in patients entering alcohol detoxification programs because they have both a high prevalence of AAD risk factors and a specific vulnerability to cognitive impairment.

The aims of this pilot study were: (1) to describe the prevalence and risk factors of AAD and AAI in inpatients admitted for alcohol detoxification; (2) to analyse the association of AA level with acute cognitive impairment in the immediate follow-up to alcohol withdrawal, as measured by the Montreal Cognitive Assessment (MoCA).

## 2. Materials and Methods

### 2.1. Subjects and Sampling Procedure

A retrospective chart review of consecutive inpatients admitted for medically monitored alcohol detoxification was conducted in the department of addiction medicine of a Parisian university hospital between January and mid-March 2020. The inclusion criteria were: diagnosis with AUD according to DSM-5 criteria [26], the initiation of an inpatient program for alcohol detoxification and having a blood AA level assessment. The exclusion criteria were: having been received from another inpatient unit, having been hospitalised in the previous 3 months (considering that possible malnutrition would have been treated), having a previous prescription for AA before admission, having a decompensated cirrhosis (Child–Pugh class B or C [27]) and presenting active neoplasia or sepsis. No minimum daily alcohol consumption threshold was set for inclusion once the patient presented an AUD and a criterion for in-hospital detoxification.

### 2.2. Assessment

Patients underwent a blood sampling at the entry to assess the nutritional status (AA, vitamins D (25-hydroxycholecalciferol), B9 (folate) and B12 (cobalamin), albumin and prealbumin). All vitamins and nutrient levels were measured by automated immunoassay, except AA, by a fully validated UV high-performance liquid chromatography method. AA was sampled in the morning, after fasting and the first day after admission using a lithium/heparin-coated tube. After sampling, the AA aliquot was immediately protected from light and refrigerated for transport. AAD was defined as <2 mg/L, AAI as 2–4.99 mg/L and normal level as ≥5.0 mg/L [22,28]. Thiamine dosage and oxidative stress status measures were not available because they were not performed in routine care.

Socio-demographic factors were collected, such as age, sex, housing, socio-professional category according to the French National Institute of Statistics and Economic Studies (INSEE) classification, and educational attainment. Substance use disorders were classified according to DSM-5 criteria [26]. 

The MoCA (range 0–30) is the most common cognitive test used to screen acute alcohol-related cognitive impairment in the immediate aftermath of alcohol withdrawal [3,29]. The assessment was carried out among native French speakers between days 10 and 13 of hospitalisation, as recommended after alcohol withdrawal was completed [30]. We used the French version of the MoCA. MoCA was performed even in the case of concurrent sedative (benzodiazepine or Z-drug) use disorder, despite the continued use of a sedative. In patients without sedative use disorder, the benzodiazepines used during monitored alcohol withdrawal were stopped for at least 48 h. MoCA score multivariate analysis is controlled for this sedative use disorder.

### 2.3. Nutritional Management

Hospitalised patients had access to nutritional care supervised by a nutritionist. They were offered balanced nutrition and therapeutic education groups. In the case of deficiency of vitamins D, B9 or B12, or protein deficiency, supplementation was introduced in the early days of hospitalisation. The result of the AA measurement in our hospital required more time and was not available before the completion of the MoCA; thus, supplementation, if required, was delayed.

### 2.4. Statistical Analysis

#### 2.4.1. Factors Associated with Ascorbic Acid Deficiency and Insufficiency

In univariate analysis, the AA categories were analysed with Fischer or Chi-square tests for categorical variables and, for continuous variables, the Kruskal–Wallis test or ANOVA. A multivariate analysis was performed with multinomial regression to identify independent factors associated with AAD or AAI, including factors associated with a *p*-value ≤ 0.05 at the univariate analysis step.

#### 2.4.2. Factors Associated with MoCA Score

We chose to test the association between MoCA scores and the continuous AA levels in this specific population rather than the scurvy diagnosis thresholds for AAD [22,28]. Indeed, there are no data showing that the plasmatic levels of scurvy-defining AAD threshold are applicable when trying to measure a cognitive impact. For this, we used a polynomial regression after observing that the conditions for the validity of the linear model were not satisfied. We also conducted a multivariate analysis to predict MoCA scores with the AA level and other clinical and biological factors selected by univariate analyses with a level for entry at *p* ≤ 0.05 (Mann–Whitney–Wilcoxon and Kruskal–Wallis tests for categorical variables and linear or polynomial regression for continuous variables as appropriate). Age, sex, sedative use disorder and cannabis use disorder were forced in the model in order to adjust for those published confounders. A sensitivity analysis of MoCA scores between the AA categories was conducted by a Kruskal–Wallis test. 

All statistical analyses were performed with R software version 3.2.

### 2.5. Ethics

The study was conducted following French laws on biomedical research (Loi Jardé 2014, décrets d’application 2017) and in adherence to the Declaration of Helsinki. The Commission Nationale Informatique et Liberté (CNIL, French National Board for Information Systems and Freedom) delivered a specific authorisation to our hospital (Number 2017–013) for the analysis of data collected during routine care. Patients were informed and could oppose the use of their anonymised routine health care data for research purposes.

## 3. Results

### 3.1. Characteristics of Subjects

One hundred and seventy-two inpatients were screened. Ninety-six patients were included, of which seventy-four were men (77.1%). The mean age was 49.1 years (SD ± 11.5), with a range of 24–79 years. The mean alcohol intake per day was 225 g (±130). Seventy-three patients (79.2%) were current smokers and 12 (12.5%) former smokers. Seventeen patients (17.7%) were street homeless, and sixteen (16.7%) were sheltered homeless. Eight patients (8.3%) were asymptomatic people living with HIV. The subjects’ characteristics are presented in Table 1. The flow chart is shown in Supplementary Materials Figure S1.

### 3.2. Prevalence of Ascorbic Acid Deficiency and Insufficiency

Twenty-seven patients presented with AAD (28.1%), 22 with AAI (22.9%) and 47 had a normal AA level (49.0%).

### 3.3. Factors Associated with Ascorbic Acid Deficiency and Insufficiency

In univariate analysis, we observed an association of AA categories with sex (*p* = 0.011), housing (*p* = 0.014) and compensated cirrhosis (*p* = 0.028). Regarding AAD, the rate is 35.1% in men (26/74) versus 4.5% in women (1/22). It is 52.9% in homeless subjects living on the streets (9/17), 31.2% in homeless subjects living in a shelter (5/16) and 61.5% in subjects with compensated cirrhosis (8/13). No significant difference was observed for alcohol intake per day (*p* = 0.20) or other co-occurring substance use disorders (*p* > 0.05). The results are presented in Table 2.

In multivariate analysis, versus normal AA level, AAD was associated with men (OR 17.8, 95%CI (1.63–194)), compensated cirrhosis (OR 9.35, 95%CI (1.60–54.6)), versus no cirrhosis) and street homelessness (OR 5.76, 95%CI (1.24–26.8) versus personal housing).

### 3.4. Factors Associated with MoCA Score

The MoCA score was available for 53 patients (55.2%) (Supplementary Materials Figure S1). Characteristics of the subsample are presented in Table 1. They differ from the whole sample in terms of age, educational attainment, alcohol intake per day, tobacco consumption (*p* < 0.001) and socio-professional category (*p* = 9.2 × 10^−3^).

**Figure 1:**
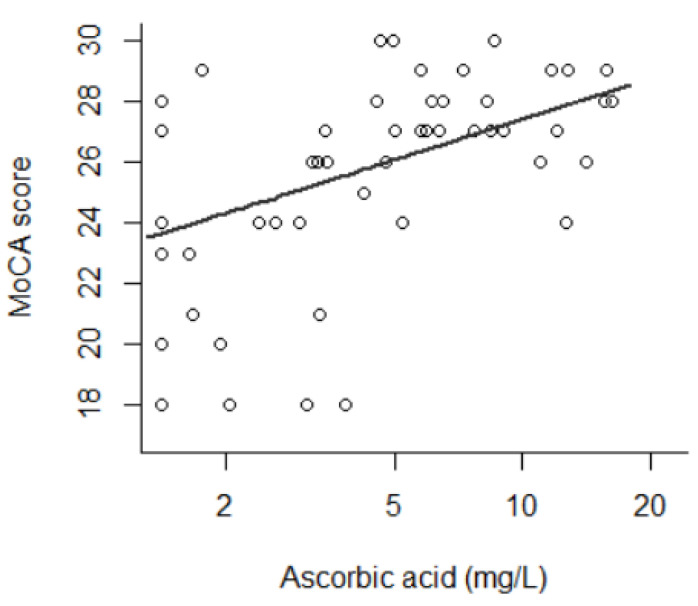
Montreal Cognitive Assessment score as a function of ascorbic acid (AA) level (β of AA natural logarithm = 1.91, *p* = 5.3 × 10^−4^) (*n* = 53). AA is presented in logarithmic range.

The average MoCA score was 25.7 (±3.3), with a range from 18 to 30. In univariate analysis, the MoCA score was associated with the natural logarithm of AA (β = 1.91, *p* = 5.3 × 10^−4^); i.e., a lower MoCA score was observed in patients with a lower AA concentration. This is shown in Figure 1. The MoCA score was associated with age as indicated by a U-shaped curve (by decade: β = −6.9, *p* = 0.013; by decade2: β = 0.64, *p* = 0.016), with prealbumin linearly (β = 8.3, *p* = 0.046), with housing (*p* = 4.4 × 10^−3^), with socio-professional category (*p* = 6.1 × 10^−3^) and with HIV status (*p* = 0.021). No difference was observed regarding alcohol intake (*p* = 0.21), body mass index (*p* = 0.95) or vitamins D (*p* = 0.96) and B9 (*p* = 0.92). These results are presented in Table 3. 

In the multivariate analysis, the MoCA score was associated with the natural logarithm of ascorbic acid concentration (β = 1.18, *p* = 0.037) and sedative use disorder (β = −2.77, *p* = 0.046) after adjusting for age, sex, prealbumin, housing, socio-professional category, HIV status and cannabis use (R^2^ = 0.597, *p* = 4.7 × 10^−4^) (Table 3). 

In the sensitivity analysis, the mean MoCA score was significantly lower in AAD (24.4 ± 3.5) and AAI (24.4 ± 3.9) than normal level (27.4 ± 1.5) (Kruskal–Wallis test, *p* = 3.4 × 10^−3^).

## 4. Discussion

In this study on AUD patients recruited during their inpatient treatment for alcohol detoxification, only half of the patients had a normal AA level. The prevalence of AAD observed was 28.1%. This prevalence of AAD was higher in men, patients living on the street and those suffering from compensated cirrhosis. An association between the AA level and the MoCA score was identified. This association persists after adjustment for the identified confounding factors. Very few studies have investigated the prevalence of AAD in AUD patients, and, to the best of our knowledge, this is the first study to identify an association between AA level and acute alcohol-related cognitive impairment. 

In this population with severe AUD undergoing inpatient detoxification and displaying several vulnerability factors, the prevalence of AAD and AAI was similar to that observed in severe AUD patients [9,10,11,31] and considerably higher than in the general population [12,13]. Two of the risk factors of AAD identified in this study, male sex and street homelessness, were also shared with non-AUD patients, although they have never been specifically described in AUD patients [12,13,22,24,25]. The association already described between cirrhosis and AAD was also confirmed [32]. This association involves altered hepatic metabolism, enhanced oxidative stress and the oxidation of AA and its disposition [33,34]. Conversely, we did not observe the previously reported association between AAD and tobacco consumption. This association was mostly reported in the general population, and we could face a ceiling effect in our sample (79.2% were current smokers). We also did not find any association between AAD and folates, although they have the same dietary origin in fresh vegetables and fruits. This may be explained by a specific risk of AA degradation due to cooking and storage conditions or by folate supplementation provided by previous thiamine pharmaceutical supplementation combined with folates.

Regarding the association of AA level and MoCA score screening for cognitive deficit, this result is original. During alcohol withdrawal, acute cognitive impairment may be observed due to the combination of several complications such as delirium tremens, Wernicke encephalopathy or detoxification itself. Usually, this impairment improves during the first two months [3]. Persistent cognitive impairments are less frequent but have a major impact on patients’ autonomy and constitute severe damage [3,6]. The observed lower MoCA in the first days of alcohol detoxification in the case of low AA levels could be explained by: (1) confounders, (2) pre-existing persistent cognitive disorders that deteriorate dietary intake due to the need for high planning skills and (3) increased neurological toxicity of alcohol withdrawal. In the brain, AA was shown to have a protective role in the mechanism of glutamate-induced cell death, thus protecting neurons against glutamate-generated reactive oxygen species and enabling the glutamate–AA heteroexchange [19,20,21]. Furthermore, AA is also an essential co-factor in the regular synthesis of many neurotransmitters, including noradrenaline and acetylcholine, and a neuromodulator of NMDA and dopamine receptors [19,20,21]. Thus, AA deficiency would impair synaptic conduction on a long-term basis. AA is also involved in myelin formation [35]. 

A further secondary decrease in AA levels during alcohol withdrawal has also been observed in one study, suggesting high catabolism of AA during withdrawal [9]. This decrease in AA level may explain the high prevalence of AAD in patients hospitalised for severe alcohol withdrawal and in intensive care [11]. This suggests that the antioxidant effect of AA is especially clinically relevant in conditions of glutamatergic neurotoxicity and oxidative stress, such as in AUD inpatients during alcohol withdrawal [36].

Regarding possible confounding factors for low MoCA score, we did not observe the previously observed association of MoCA score with low body mass index [1], and this could be explained by many other dietary confounding factors and differences between malnutrition subtypes (calories, proteins or vitamins intakes) [7,8,16,37].

In the univariate analysis, an HIV-positive status was associated with a lower MoCA score. Cognitive impairment in patients living with HIV is known but is usually manifested in the symptomatic stages of AIDS [38]. This association was not found in multivariate analysis after adjustment with cofounders. In the multivariate analysis, a lower MoCA score in patients with sedative use disorder was also found, which is known to affect cognitive performance [30].

In this study, patients’ usual diet prior to admission, e.g., AA-rich food intakes, was not recorded. The prevalence of AAD is related to deprivation in dietary intake [9,10,13,39], favoured by homelessness. Unfortunately, an exhaustive inventory of dietary intake is difficult in clinical practice, and this methodology has several limitations: differences in absorption and metabolism between patients, inaccuracies between food diaries and nutrient database and loss of AA during cooking and storage [14,37]. Obviously, we could not assess it retrospectively in these severe AUD patients. We acknowledge that we focused on the AA level and did not record clinical signs of scurvy. Indeed, these are unspecific and consist of asthenia, myalgia and dental and dermatological lesions that may occur for other reasons in patients with AUD [10,11,31]. Other interesting variables were not recorded, e.g., type of preferred alcohol, the pattern of consumption, delay since last drinking and alcohol withdrawal severity using validated scales or thiamine level.

Our study has some other limitations, including the retrospective chart review design, open-label study without a control group or blind record study, giving way to non-specific care setting effects. No minimum daily alcohol consumption threshold was set for inclusion. No oxidative stress status measures were performed, such as the assessment of the reactive oxygen species, their induced modifications of lipids, DNA and proteins, nor the total antioxidant capacity [40]. There was no prospective monitoring of the AA level evolution during the first few days of alcohol withdrawal and before the MoCA was completed. Finally, AA blood measurement is subject to well-known pre-analytical fluctuation due to its antioxidant nature and high lability [37].

The main strength of our study is the well-defined population, with verified AUD criteria, high mean alcohol consumption levels, the recruitment of precarious patients and/or those with complications, which allowed us to study and control for those clinical factors. The second strength is the prevalence of acute alcohol-related cognitive impairment and its routine screening process using the MoCA.

## 5. Conclusions

Our pilot study opens important perspectives. The prevalence of AAD was high. AAD increases the risk of cardiovascular diseases, infectious diseases or cancer. Moreover, in the specific domain of cognitive impairment that we explored [14,15], we found an association between AA level and early cognitive impairment. This link is supported by pharmacological data on the cerebral metabolism of AA. These cognitive impairments are prevalent and have a strong impact on the rehabilitation of patients [3]. According to experts’ opinions, the treatment of scurvy consists of per os administration of AA at a dose of 300 mg to 1 g per day, in divided doses for two weeks [41,42]. The dose could even safely be up to 2 g per day [43]. Contraindications to the prescription of AA supplementation are limited (glucose-6-phosphate dehydrogenase deficiency, oxalosis, gout). Side effects are predominantly digestive symptoms, nausea and diarrhoea [18,42,43]. Those points may justify an increased AA intake for all patients admitted for alcohol detoxification. Today, the adequate dose and route of administration for pharmaceutical AA supplementation in the general population are debated [18,19]. In order to reach the recommended grade of systematic thiamine supplementation in alcohol withdrawal, universal AA supplementation during alcohol detoxification should be evaluated. A clinical trial assessing AA supplementation in the form of pharmaceutical preparation or a specific diet enriched in fruits and vegetables versus placebo is warranted in this specific population.

## Figures and Tables

**Table 1:** Subjects’ characteristics (*n* = 96).

		All Patients	Subsample with Available MoCA (*n* = 53)
		(*n* = 96)	
Sex	Women	22 (22.9%)	12 (22.6%)
	Men	74 (77.1%)	41 (77.4%)
Age (years)	Mean (SD)	49.1 (±11.5)	50.2 (±11.2)
	Min–Max	24–79	29–79
Body mass index (kg/m^2^) (*n* = 91)	<21.0	19 (20.7%)	10 (19.2%)
	21.0–24.99	43 (46.7%)	27 (51.9%)
	≥25.0	30 (32.6%)	15 (28.8%)
Housing	Private home	63 (65.6%)	39 (73.6%)
	Homeless in a shelter	16 (16.7%)	9 (17.0%)
	Street homelessness	17 (17.7%)	5 (9.4%)
Educational attainment (*n* = 94)	≥Bachelor’s degree	28 (29.8%)	25 (47.2%)
	High school degree	23 (24.5%)	11 (20.8%)
	<High school degree	43 (45.7%)	17 (32.1%)
Socio-professional category *	Higher	24 (25.0%)	18 (34.0%)
	Intermediate	12 (12.5%)	9 (17.0%)
	Lower	60 (62.5%)	26 (49.0%)
Type of admission	Planned	77 (80.2%)	46 (86.8%)
	Via the Emergency Department	19 (19.8%)	7 (13.2%)
Alcohol intake per day (grams) (*n* = 95)	Mean (SD)	225 (±130)	212 (±108)
	Min–Max	20–600	64–550
Tobacco use	Current smoker	76 (79.2%)	42 (79.2%)
	Former smoker	12 (12.5%)	7 (13.2%)
	Non-smoker	8 (8.3%)	4 (7.5%)
Cigarettes per day (*n* = 93)	Mean (SD)	15.0 (±12.7)	16.1 (±13.8)
Number of tobacco pack years (*n* = 84)	Mean (SD)	31.4 (±17.9)	32.1 (±15.9)
Number of years of tobacco smoking (*n* = 88)	Mean (SD)	28.6 (±13.5)	30.4 (±13.1)
Cirrhosis	Compensated	13 (13.5%)	9 (17.0%)
	No cirrhosis	83 (86.5%)	44 (83.0%)
HIV status	Yes	8 (8.3%)	5 (9.4%)
	No	88 (91.7%)	48 (90.6%)
Hypertension	Yes	11 (11.5%)	8 (15.1%)
	No	85 (88.5%)	45 (85.9%)
Cannabis use disorder **	Yes	20 (20.8%)	12 (22.6%)
	No	76 (79.2%)	41 (77.4%)
Sedative use disorder **	Yes	12 (12.5%)	6 (11.3%)
	No	84 (87.5%)	47 (88.7%)
Cocaine use disorder **	Yes	13 (13.5%)	4 (7.5%)
	No	83 (86.5%)	49 (92.5%)
Opiate use disorder **	Active	2 (2.1%)	0 (0%)
	Opioid maintenance treatment	10 (10.4%)	3 (5.7%)
	No	84 (87.5%)	50 (94.7%)
Psychiatric comorbidity	Bipolar disorder	4 (4.2%)	2 (3.8%)
	Schizophrenia	2 (2.1%)	1 (1.9%)
Selective serotonin reuptake inhibitor current use	Yes	33 (34.4%)	24 (45.3%)
	No	63 (65.6%)	29 (54.7%)

* Socio-professional category according to INSEE classification. ** Substance use disorder according to DSM-5 criteria.

**Table 2:** Factors associated with ascorbic acid categories (*n* = 96).

		Ascorbic Acid Level (mg/L)			
		Deficiency (<2.0)	Insufficiency (2.0–4.99)	Normal Level (≥5.0)	*p*
Sex	Women	1 (4.5%)	5 (22.7%)	16 (72.7%)	0.011 ^††^
	Men	26 (35.1%)	17 (23.0%)	31 (41.9%)	
Age (years)	Mean (SD)	46.7 (±10.1)	49.4 (±10.8)	50.3 (±12.5)	0.43 ^†††^
Body mass index (kg/m^2^)	<21.0	5 (26.3%)	3 (15.8%)	11 (57.9%)	0.93 ^†^
	21.0–24.99	11 (25.6%)	11 (25.6%)	21 (48.8%)	
	≥25.0	8 (26.7%)	8 (26.7%)	14 (46.7%)	
Prealbumin (g/L)	Mean (SD)	0.24 (±0.11)	0.29 (±0.11)	0.28 (±0.10)	0.23 ^†††^
Albumin (g/L)	Mean (SD)	40.2 (±5.3)	42.5 (±3.8)	42.5 (±3.9)	0.074 ^†††^
Vitamin D	<10 ng/L	19 (36.5%)	13 (25.0%)	20 (38.5%)	0.28 ^†^
	10–20 ng/L	4 (19.0%)	4 (19.0%)	13 (61.9%)	
	≥20 ng/L	4 (18.2%)	5 (22.7%)	13 (59.1%)	
Vitamin B9	<3.9 µg/L	7 (33.3%)	6 (28.6%)	8 (38.1%)	0.50 ^†^
	≥3.9 µg/L	20 (26.7%)	16 (21.3%)	39 (52.0%)	
Vitamin B12	<197 ng/L	0 (0%)	1 (50.0%)	1 (50.0%)	0.47 ^†^
	≥197 ng/L	27 (21.5%)	20 (29.0%)	46 (49.5%)	
Housing	Private home	13 (20.6%)	12 (19.0%)	38 (60.3%)	0.014 ^†^
	Homeless in a shelter	5 (31.2%)	5 (31.2%)	6 (37.5%)	
	Street homelessness	9 (52.9%)	5 (29.4%)	3 (17.6%)	
Academic attainment	≥Bachelor’s degree	6 (21.4%)	6 (21.4%)	16 (57.1%)	0.24 ^†^
	High school degree	3 (13.0%)	6 (29.1%)	14 (60.9%)	
	<High school degree	16 (37.2%)	10 (23.3%)	17 (39.5%)	
Socioprofessional category *	Higher	5 (20.8%)	3 (12.5%)	16 (66.7%)	0.23 ^†^
	Intermediate	2 (16.7%)	3 (25%)	7 (58.3%)	
	Lower	20 (33.3%)	16 (26.7%)	24 (40.0%)	
Type of admission	Planned	18 (23.4%)	18 (23.4%)	41 (53.2%)	0.13 ^†^
	Urgently	9 (47.4%)	4 (21.1%)	6 (31.6%)	
Alcohol intake per day (grams)	Mean (SD)	248 (±136)	251 (±139)	198 (±119)	0.20 ***
	Min–Max	60–600	80–600	20–600	
Tobacco use	Current smoker	23 (30.3%)	17 (22.4%)	36 (47.4%)	0.72 ^†^
	Former or non-smoker	4 (20.0%)	5 (25.0%)	11 (55.0%)	
Cigarettes per day	Mean (SD)	15.2 (±12.3)	17.4 (±13.7)	13.8 (±12.6)	0.51 ***
Number of tobacco pack years	Mean (SD)	27.8 (±15.2)	33.5 (±16.0)	32.5 (±20.3)	0.55 ***
Number of years of tobacco smoking	Mean (SD)	27.0 (±13.1)	29.5 (±11.1)	29.1 (±14.9)	0.73 ***
Cirrhosis	Compensated	8 (61.5%)	1 (7.7%)	4 (30.8%)	0.028 ^†^
	No cirrhosis	19 (22.9%)	21 (25.3%)	43 (51.8%)	
HIV status	Yes	4 (50.0%)	2 (25.0%)	2 (25.0%)	0.27 ^†^
	No	23 (26.1%)	20 (22.7%)	45 (51.1%)	
Cannabis use disorder **	Yes	2 (10.0%)	5 (25.0%)	13 (65.0%)	0.099 ^†^
	No	25 (22.4%)	17 (32.9%)	34 (44.7%)	
Sedative use disorder **	Yes	2 (16.7%)	3 (25.0%)	7 (58.3%)	0.71 ^†^
	No	25 (29.8%)	19 (22.6%)	40 (47.6%)	
Cocaine use disorder **	Yes	2 (15.4%)	3 (23.1%)	8 (61.5%)	0.54 ^†^
	No	25 (30.1%)	19 (22.9%)	39 (47.0%)	
Opiate use disorder **	Active	0	0	2 (100%)	0.21 ^†^
	Opioid maintenance treatment	5 (55.6%)	2 (22.2%)	2 (22.2%)	
	No	22 (25.9%)	20 (23.5%)	43 (50.6%)	

* Socio-professional category according to INSEE classification. ** Substance use disorder according to DSM-5 criteria. *** Kruskal–Wallis test. ^†^ Fisher test, ^††^ Chi-square test, ^†††^ ANOVA.

**Table 3:** Factors associated with Montreal Cognitive Assessment score at days 10–13 (*n* = 53).

		Univariate Analysis		Multivariate Analysis *** (*n* = 47)	
		MoCA Score	*p*-Value	β	*p*-Value
Ascorbic acid	natural logarithm (mg/L)	β = 1.91	5.3 × 10^−4 †††^	1.18	0.037
Sex	Women	27.2 (±1.8)	0.15 ^††^	ref	0.54
	Men	25.3 (±3.5)		−0.53	
Age (years)	/decade	β = −6.9	0.013 ***	−4.1	0.099
	/decade^2^	β’ = 0.64	0.016 ***	0.31	0.19
Prealbumin (g/L) (*n* = 47)		β = 8.3	0.046 ^†††^	2.50	0.51
Albumin (g/L)		β = 0.10	0.37 ^†††^	-	-
Vitamin D (ng/L) (*n* = 52)		β = −1.7 × 10^−3^	0.96 ^†††^	-	-
Vitamin B9 (µg/L)		β = −0.014	0.92 ^†††^	-	-
Vitamin B12 (ng/L) (*n* = 52)		β = 5.6 × 10^−4^	0.84 ^†††^	-	-
Body mass index (kg/m^2^) (*n* = 52)	<21.0	25.7 (±2.9)	0.95 ^††^	-	-
	21.0–24.99	25.7 (±3.5)			
	≥25.0	25.8 (±3.4)			
Housing	Private home	26.5 (±2.7)	4.4 × 10^−3 ††^	ref	ref
	Homeless in a shelter	25.2 (±3.4)		1.29	0.19
	Street homelessness	20.1 (±2.5)		−2.01	0.17
Socioprofessional category *	Higher	27.0 (±2.4)	6.1 × 10^−3 ††^	ref	ref
	Intermediate	26.0 (±3.3)		1.23	0.29
	Lower	24.1 (±3.6)		−1.69	0.053
Alcohol intake	by 10 g per day	β = −0.052	0.21 ^†††^	-	-
Tobacco use	Current smoker	25.4 (±3.4)	0.98 ^†^	-	-
	Former or non-smoker	25.5 (±3.7)			
Cigarettes (*n* = 52)	Number use per day	β = 0.014	0.69 ^†††^	-	-
Cirrhosis	Compensated	24.9 (±4.3)	0.78 ^†^	-	-
	No cirrhosis	25.9 (±3.1)			
HIV status	Yes	22.0 (±3.5)	0.021 ^†^	−2.67	0.075
	No	26.1 (±3.0)			
Cannabis use disorder **	Yes	26.6 (±3.3)	0.35 ^†^	0.72	0.50
	No	25.5 (±3.3)		ref	
Sedative use disorder **	Yes	24.8 (±3.6)	0.35 ^†^	−2.77	0.046
	No	25.8 (±3.3)		ref	

^†^ Mann–Whitney–Wilcoxon, ^††^ Kruskal–Wallis test, ^†††^ Linear regression. * Socio-professional category according to INSEE classification, ** Substance use disorder according to DSM-5 criteria, *** Polynomial regression (R^2^ = 0.597, *p* = 4.7 × 10^−4^).

## Data Availability

Data available upon request due to ethical reasons. The data are the property of the APHP.

## Supplementary Materials

The following are available online at https://www.mdpi.com/article/10.3390/antiox10121892/s1, Figure S1: Flow chart.

## References

[1] Gautron M.-A., Questel F., Lejoyeux M., Bellivier F., Vorspan F. (2017). Nutritional Status during Inpatient Alcohol Detoxification. Alcohol Alcohol..

[2] Coulbault L., Ritz L., Vabret F., Lannuzel C., Boudehent C., Nowoczyn M., Beaunieux H., Pitel A.L. (2021). Thiamine and phosphate esters concentrations in whole blood and serum of patients with alcohol use disorder: A relation with cognitive deficits. Nutr. Neurosci..

[3] Rao R., Topiwala A. (2020). Alcohol use disorders and the brain. Addiction.

[4] Luquiens A., Rolland B., Pelletier S., Alarcon R., Donnadieu-Rigole H., Benyamina A., Nalpas B., Perney P. (2019). Role of Patient Sex in Early Recovery from Alcohol-Related Cognitive Impairment: Women Penalized. J. Clin. Med..

[5] Maillard A., Poussier H., Boudehent C., Lannuzel C., Vicente A., Vabret F., Cabe N., Pitel A.-L. (2020). Short-term neuropsychological recovery in alcohol use disorder: A retrospective clinical study. Addict. Behav..

[6] McLean C., Tapsell L., Grafenauer S., McMahon A.T. (2020). Systematic review of nutritional interventions for people admitted to hospital for alcohol withdrawal. Nutr. Diet..

[7] Addolorato G., Capristo E., Stefanini G.F., Gasbarrini G. (1998). Metabolic Features and Nutritional Status in Chronic Alcoholics. Am. J. Gastroenterol..

[8] Ross L.J., Wilson M., Banks M., Rezannah F., Daglish M. (2012). Prevalence of malnutrition and nutritional risk factors in patients undergoing alcohol and drug treatment. Nutrition.

[9] LeComte E., Herbeth B., Pirollet P., Chancerelle Y., Arnaud J., Musse N., Paille F., Siest G., Artur Y. (1994). Effect of alcohol consumption on blood antioxidant nutrients and oxidative stress indicators. Am. J. Clin. Nutr..

[10] Lux-Battistelli C., Battistelli D. (2019). Alcohol Withdrawal: Possible Risk of Latent Scurvy Appearing as Tiredness: A STROBE-Compliant Study. J. Clin. Med. Res..

[11] Marik P.E., Liggett A. (2019). Adding an orange to the banana bag: Vitamin C deficiency is common in alcohol use disorders. Crit. Care.

[12] Schleicher R.L., Carroll M.D., Ford E.S., A Lacher D. (2009). Serum vitamin C and the prevalence of vitamin C deficiency in the United States: 2003–2004 National Health and Nutrition Examination Survey (NHANES). Am. J. Clin. Nutr..

[13] Galan P., Viteri F.E., Bertrais S., Czernichow S., Faure H., Arnaud J., Ruffieux D., Chenal S., Arnault N., Favier A. (2005). Serum concentrations of β-carotene, vitamins C and E, zinc and selenium are influenced by sex, age, diet, smoking status, alcohol consumption and corpulence in a general French adult population. Eur. J. Clin. Nutr..

[14] Morelli M.B., Gambardella J., Castellanos V., Trimarco V., Santulli G. (2020). Vitamin C and Cardiovascular Disease: An Update. Antioxidants.

[15] Berretta M., Quagliariello V., Maurea N., Di Francia R., Sharifi S., Facchini G., Rinaldi L., Piezzo M., Manuela C., Nunnari G. (2020). Multiple Effects of Ascorbic Acid against Chronic Diseases: Updated Evidence from Preclinical and Clinical Studies. Antioxidants.

[16] Scarmeas N., Anastasiou C.A., Yannakoulia M. (2018). Nutrition and prevention of cognitive impairment. Lancet Neurol..

[17] Travica N., Ried K., Sali A., Scholey A., Hudson I., Pipingas A. (2017). Vitamin C Status and Cognitive Function: A Systematic Review. Nutrients.

[18] Carr A.C., Lykkesfeldt J. (2021). Discrepancies in global vitamin C recommendations: A review of RDA criteria and underlying health perspectives. Crit. Rev. Food Sci. Nutr..

[19] Harrison F.E., Bowman G.L., Polidori M.C. (2014). Ascorbic Acid and the Brain: Rationale for the Use against Cognitive Decline. Nutrients.

[20] Ballaz S., Morales I., Rodríguez M., Obeso J.A. (2013). Ascorbate prevents cell death from prolonged exposure to glutamate in an in vitro model of human dopaminergic neurons: Ascorbate Prevents Cell Death from Glutamate. J. Neurosci. Res..

[21] Lykkesfeldt J., Tveden-Nyborg P. (2019). The Pharmacokinetics of Vitamin C. Nutrients.

[22] Fain O., Pariés J., Jacquart B., Le Moël G., Kettaneh A., Stirnemann J., Héron C., Sitbon M., Taleb C., Letellier E. (2003). Hypovitaminosis C in hospitalized patients. Eur. J. Intern. Med..

[23] Sharma Y., Miller M., Shahi R., Doyle A., Horwood C., Hakendorf P., Thompson C. (2019). Vitamin C deficiency in Australian hospitalised patients: An observational study: Vitamin C deficiency. Intern. Med. J..

[24] Mosdøl A., Erens B., Brunner E. (2008). Estimated prevalence and predictors of vitamin C deficiency within UK’s low-income population. J. Public Health.

[25] Malmauret L., Leblanc J., Cuvelier I., Verger P. (2002). Dietary intakes and vitamin status of a sample of homeless people in Paris. Eur. J. Clin. Nutr..

[26] American Psychiatric Association (2013). Diagnostic and Statistical Manual of Mental Disorders.

[27] Cholongitas E., Papatheodoridis G., Vangeli M., Terreni N., Patch D., Burroughs A.K. (2005). Systematic review: The model for end-stage liver disease—should it replace Child-Pugh’s classification for assessing prognosis in cirrhosis?. Aliment. Pharmacol. Ther..

[28] Johnston C.S., Thompson L.L. (1998). Vitamin C status of an outpatient population. J. Am. Coll. Nutr..

[29] Heirene R., John B., Roderique-Davies G. (2018). Identification and Evaluation of Neuropsychological Tools Used in the Assessment of Alcohol-Related Cognitive Impairment: A Systematic Review. Front. Psychol..

[30] Alarcon R., Nalpas B., Pelletier S., Perney P. (2015). MoCA as a Screening Tool of Neuropsychological Deficits in Alcohol-Dependent Patients. Alcohol. Clin. Exp. Res..

[31] Lee H.J., Shin J., Hong K.J., Jung J.H. (2015). Vitamin C Deficiency of Korean Homeless Patients Visiting to Emergency Department with Acute Alcohol Intoxication. J. Korean Med. Sci..

[32] Beattie A.D., Sherlock S. (1976). Ascorbic acid deficiency in liver disease. Gut.

[33] Clot P., Tabone M., Arico S., Albano E. (1994). Monitoring oxidative damage in patients with liver cirrhosis and different daily alcohol intake. Gut.

[34] Hernandez-Guerra M., Garcia-Pagan J.C., Turnes J., Bellot P., Deulofeu R., Abraldes J.G., Bosch J. (2006). Ascorbic acid improves the intrahepatic endothelial dysfunction of patients with cirrhosis and portal hypertension. Hepatology.

[35] Guo Y.-E., Suo N., Cui X., Yuan Q., Xie X. (2018). Vitamin C promotes oligodendrocytes generation and remyelination. Glia.

[36] Zhang X.-Y., Xu Z.-P., Wang W., Cao J.-B., Fu Q., Zhao W.-X., Li Y., Huo X.-L., Zhang L.-M., Li Y.-F. (2018). Vitamin C alleviates LPS-induced cognitive impairment in mice by suppressing neuroinflammation and oxidative stress. Int. Immunopharmacol..

[37] Michels A., Frei B. (2013). Myths, Artifacts, and Fatal Flaws: Identifying Limitations and Opportunities in Vitamin C Research. Nutrients.

[38] Valcour V., Paul R., Chiao S., Wendelken L.A., Miller B. (2011). Screening for Cognitive Impairment in Human Immunodeficiency Virus. Clin. Infect. Dis..

[39] Hercberg S., Preziosi P., Galan P., Devanlay M., Keller H., Bourgeois C., De Courcy G.P., Cherouvrier F. (1994). Vitamin status of a healthy French population: Dietary intakes and biochemical markers. Int. J. Vitam. Nutr. Res..

[40] Marrocco I., Altieri F., Peluso I. (2017). Measurement and Clinical Significance of Biomarkers of Oxidative Stress in Humans. Oxidative Med. Cell. Longev..

[41] Smith A., Di Primio G., Humphrey-Murto S. (2011). Scurvy in the developed world. Can. Med. Assoc. J..

[42] Fain O. (2004). Carences en vitamine C. La Rev. de Médecine Interne.

[43] Institute of Medicine (US) (2000). Dietary Reference Intakes for Vitamin C, Vitamin E, Selenium, and Carotenoids.

