# Supplementary material for: Ascorbic Acid Deficiency Prevalence and Associated Cognitive Impairment in Alcohol Detoxification Inpatients: A Pilot Study
Source: Antioxidants (Basel). 2021 Nov 26;10(12):1892. doi: 10.3390/antiox10121892 (PMC8750659; doi:10.3390/antiox10121892)

Ascorbic acid deficiency prevalence and associated cognitive impairment in alcohol detoxification inpatients: A pilot study

SUPPLEMENTARY MATERIALS

Supplementary Materials Figure S1: Flow chart

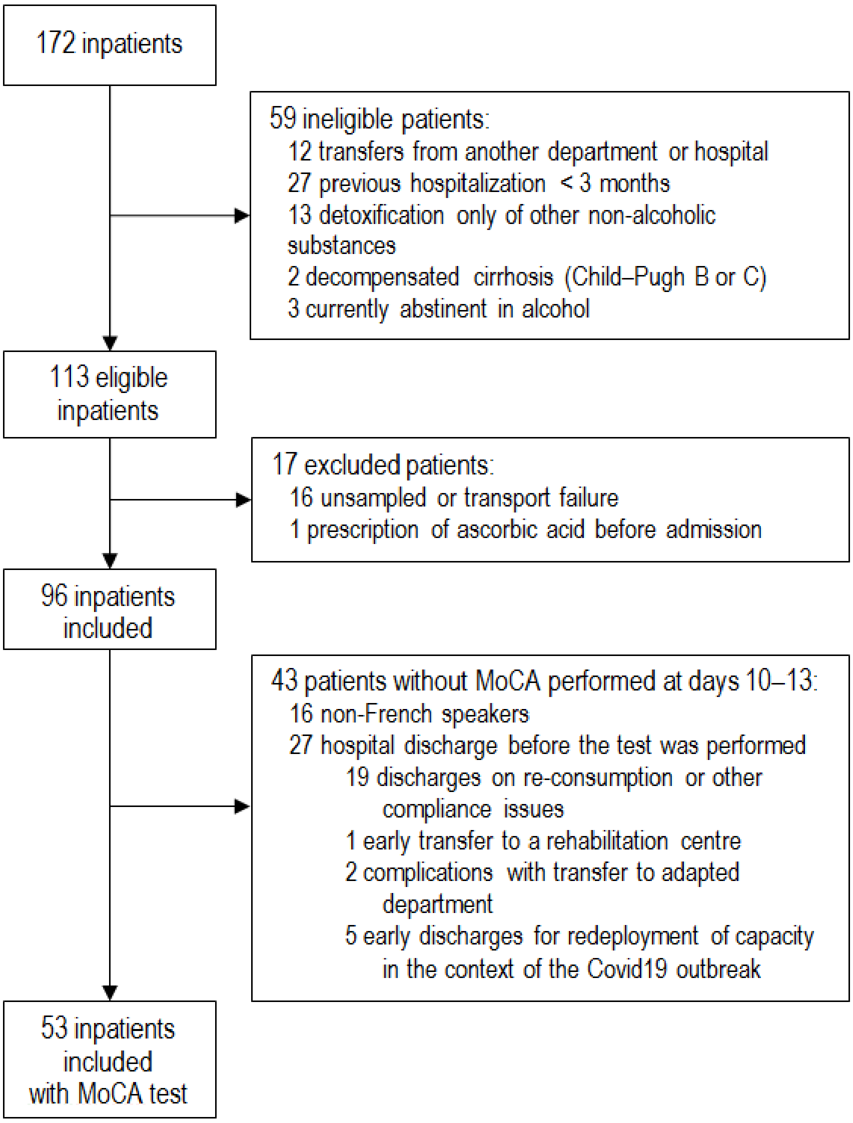

Supplement: Supplementary file 1 [file antioxidants-10-01892-s001.zip › antioxidants-1456895-supplementary.pdf]
